# Genomic and phenotypic adaptation of imipenem-resistant *Pseudomonas aeruginosa* during chronic infection in a COPD patient

**DOI:** 10.1128/spectrum.01358-26

**Published:** 2026-08-20

**Authors:** Lvxin Qian, Yi Yan, Shuai Sun, Junren Zeng, Furong Zhang, Li Xiang, Huan Chen, Luhua Zhang, Ying Li

**Affiliations:** 1 The School of Basic Medical Sciences, Southwest Medical University 74647, Luzhou, Sichuan, China; 2 Department of Clinical Laboratory, Pengzhou People’s Hospital, Chengdu, Sichuan, China; University of Manitoba, Winnipeg, Manitoba, USA

**Keywords:** *P. aeruginosa*, imipenem-resistant, mucoid, non-mucoid, adaptive

## Abstract

*Pseudomonas aeruginosa* airway colonization and infection are major drivers of chronic obstructive pulmonary disease (COPD) acute exacerbations, yet its adaptive evolution in the COPD airway remains poorly understood. Here, six serial *P. aeruginosa* strains were isolated over a 5-month interval from a single COPD patient: two initial isolates (SCPa03-1 and SCPa03-2) from one sputum sample and four subsequent isolates (SCPa12-1, SCPa12-2, SCPa12-3, and SCPa12-4) from another. Of them, SCPa03-1, SCPa12-1, and SCPa12-2 exhibited a non-mucoid phenotype, while SCPa03-2, SCPa12-3, and SCPa12-4 were mucoid. All isolates were resistant to most *β*-lactam antibiotics, including imipenem, but susceptible to amikacin and gentamicin. Notably, three mucoid strains were susceptible to meropenem. Genomic and phylogenetic analysis revealed that all isolates were clonally related, belonged to ST274, and were closely related to the globally prevalent high-risk ST274 clone. Sequence analysis and qRT-PCR suggested that their imipenem resistance may be associated with *ampC* overexpression and *oprD* mutations, while reduced ciprofloxacin susceptibility likely stemmed from *gyrA*/*gyrB* mutations and acquisition of *crpP*-harboring ICEs. Despite a shared *mucA* frameshift mutation, secondary mutations in *prc* (SCPa03-1) and *algU* (SCPa12-1, SCPa12-2) likely accounted for the non-mucoid phenotype. Relative to PAO1, all isolates showed reduced growth, diminished pyoverdine, attenuated *Galleria mellonella* virulence, and increased pyocyanin. Besides, three mucoid strains exhibited slower growth, reduced serum resistance, and attenuated virulence compared with non-mucoid counterparts, suggesting adaptive trade-off during host adaptation. Four late-stage isolates showed enhanced pyoverdine production and improved growth under iron limitation, indicating progressive airway adaptation to the iron-restricted airway niche.

**IMPORTANCE:**

*Pseudomonas aeruginosa* is a leading cause of chronic airway infection and poses a severe clinical threat when it develops carbapenem resistance. While the adaptive evolution of *P. aeruginosa* has been well characterized in cystic fibrosis, its evolutionary patterns during chronic infection in patients with chronic obstructive pulmonary disease (COPD) remain poorly understood. Here, we analyzed six imipenem-resistant *P. aeruginosa* isolates sequentially recovered from a single COPD patient. Integrated genomic and phenotypic analyses demonstrated that the high-risk clone ST274 diversified extensively within the host, with genetic variations strongly associated with enhanced airway persistence. These findings advance our understanding of how carbapenem-resistant *P. aeruginosa* adapts during chronic COPD infection and provide new insights into resistance evolution and adaptive strategies that may inform the control of drug-resistant chronic respiratory infections.

## INTRODUCTION

*Pseudomonas aeruginosa* (*P. aeruginosa*) is a major gram-negative opportunistic pathogen that exhibits a remarkable capacity for airway colonization and microenvironmental adaptation during chronic respiratory tract infections (1). Currently, carbapenem-resistant *P. aeruginosa* has been listed in the “high-priority” category of the World Health Organization (WHO) Bacterial Priority Pathogens List, underscoring its significant threat to global public health (2). Notably, several internationally disseminated high-risk clones of *P. aeruginosa*, including sequence type 274 (ST274), have been increasingly associated with multidrug resistance and persistent clinical infections (3, 4). Chronic obstructive pulmonary disease (COPD) is a progressive chronic respiratory disease characterized by persistent airflow limitation and chronic airway inflammation, constituting a major global public health burden (5, 6). In patients with COPD, airway colonization of *P. aeruginosa* is closely linked to increased frequency of acute exacerbations, higher hospitalization rates, and adverse clinical outcomes, representing a key pathogenic factor that accelerates progression of the disease (7, 8).

During chronic infections, *P. aeruginosa* exhibits extensive adaptive potential, characterized by prominent genomic diversity and phenotypic variation, together with the coordinated remodeling of quorum sensing, virulence regulation, immune evasion, and antimicrobial resistance (9). The transition to the mucoid phenotype in *P. aeruginosa* is widely regarded as a hallmark of chronic infection and is associated with disease progression (10). Concurrently, the virulence strategies of *P. aeruginosa* shift from acute virulence phenotypes (such as the type III secretion system, motility, and toxin production) to chronic virulence phenotypes that facilitate long-term colonization (10). In addition, iron limitation resulting from host-mediated nutritional immunity is regarded as a key selective pressure that drives adaptive evolution of *P. aeruginosa* and induces the remodeling of its iron acquisition systems (11, 12). The siderophore pyoverdine plays a central role in iron uptake and displays dynamic regulatory features during chronic airway infection (13). Collectively, these adaptive trajectories reflect pathogen-specific evolutionary strategies shaped by sustained host selective pressures.

Current studies on the within-host adaptive evolution of *P. aeruginosa* have been primarily focused on patients with cystic fibrosis (CF), whose clinical isolates commonly exhibit well-defined adaptive traits including a mucoid phenotype, slow growth, and loss of motility (14). By contrast, studies on the adaptive evolution of *P. aeruginosa* within COPD airway microenvironments remain relatively limited. Although COPD shares core features of chronic airway inflammation and impaired host defense with CF, it displays a relatively lower mucus viscosity in the airway microenvironment, as well as distinct differences in inflammatory profiles and microbial community structure (15). Variations in host microenvironmental conditions are recognized as a key driver of genetic and phenotypic alterations in *P. aeruginosa*. Therefore, genetic evolutionary characteristics and associated phenotypic changes of *P. aeruginosa* in COPD remain to be systematically elucidated.

In this study, we performed comprehensive genomic and phenotypic characterization of six imipenem-resistant ST274 *P. aeruginosa* isolates that were obtained from two sputum samples collected 5 months apart from a COPD patient. By integrating whole-genome sequencing (WGS) with extensive phenotypic analyses, we delineated the within-host adaptive evolutionary dynamics of *P. aeruginosa* during chronic COPD infection, providing new insights into the pathogen’s adaptive strategies in this clinically important disease context.

## RESULTS

### Bacterial isolation and phenotypic characterization

In November 19, 2023, a 70-year-old man was diagnosed with chronic obstructive pulmonary disease with acute lower respiratory tract infection and was admitted to the Pengzhou People’s Hospital in Sichuan province, China. Two *P. aeruginosa* strains, SCPa03-1 and SCPa03-2, were isolated from the same sputum sample 2 days after hospital admission. During hospitalization, the patient received antibiotics piperacillin/tazobactam and amikacin for anti-infection treatment. The patient showed improvement in their overall condition and was discharged on the 15th day of admission. The patient was hospitalized again on April 14, 2024, also due to chronic obstructive pulmonary disease with acute lower respiratory tract infection. Four *P. aeruginosa* strains (SCPa12-1, SCPa12-2, SCPa12-3, and SCPa12-4) with distinct morphotypes were isolated from the same sputum sample. On admission, the patient was treated with amikacin for anti-infection treatment. After 10 days of treatment, the patient’s condition improved and was discharged from the hospital.

**Fig 1:**
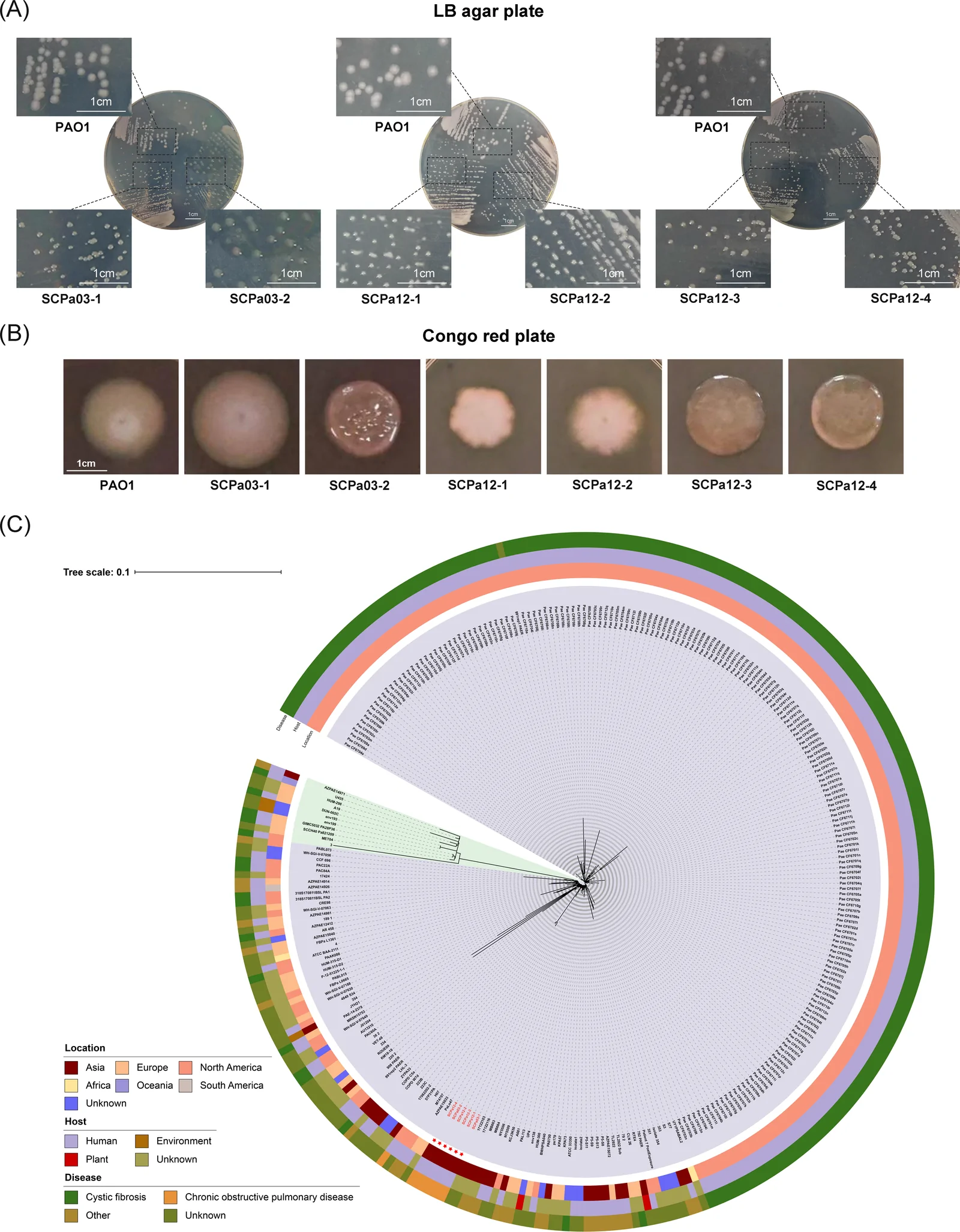
Phenotypic and phylogenetic characteristics of six *P. aeruginosa* isolates. (**A**) Colony morphology of six *P. aeruginosa* isolates after 36 h of growth at 37°C on LB agar plates. Boxes indicate areas of increased magnification. The 1 cm scale is indicated. (**B**) Colony morphology of six *P. aeruginosa* isolates after 24 h of incubation at room temperature on Congo red agar plates. The 1 cm scale is indicated. (**C**) Phylogenetic relationship of the six *P. aeruginosa* isolates (highlighted in red) with 293 globally prevalent ST274 strains. Strains in gray belong to Group A, while those in light green belong to Group B. From inside to outside, the concentric rings indicate the geographic location, host source, and associated disease of each strain, respectively.

Colony morphology analysis on Luria-Bertani (LB) agar revealed that SCPa03-2 formed large, watery colonies, whereas the other five isolates produced smaller colonies (Fig. 1A). When spotted onto Congo red agar, SCPa03-1, SCPa12-1, and SCPa12-2 displayed a non-mucoid morphology, while SCPa03-2, SCPa12-3, and SCPa12-4 exhibited mucoid morphology (Fig. 1B). Antimicrobial susceptibility testing demonstrated that all six clinical isolates were resistant to aztreonam, imipenem, ceftazidime, and cefotaxime (Table 1). SCPa03-1, SCPa12-1, and SCPa12-2 additionally showed resistance to meropenem. For ciprofloxacin and piperacillin/tazobactam, SCPa12-1 and SCPa12-3 exhibited intermediate susceptibility to the former, SCPa03-2 to the latter, and the remaining isolates were resistant to both antimicrobials. All the isolates were susceptible to amikacin and gentamicin (Table 1).

**TABLE 1:** Antimicrobial susceptibility of *P. aeruginosa* strains

Strains	Resistance phenotype^*a*^^,^^*b*^								
	ATM	IPM	MEM	CIP	CTX	CAZ	AMK	CEN	TZP
SCPa03-1	R (0)	R (11)	R (15)	R (12)	R (0)	R (0)	S (22)	S (27)	R (0)
SCPa03-2	R (15)	R (14)	S (24)	R (12)	R (0)	R (7)	S (26)	S (36)	I (0)
SCPa12-1	R (9)	R (0)	R (16)	I (22)	R (0)	R (0)	S (25)	S (31)	R (0)
SCPa12-2	R (8)	R (0)	R (12)	R (0)	R (0)	R (0)	S (25)	S (29)	R (0)
SCPa12-3	R (12)	R (13)	S (22)	I (24)	R (0)	R (0)	S (28)	S (34)	R (10)
SCPa12-4	R (12)	R (12)	S (19)	R (10)	R (7)	R (9)	S (26)	S (34)	R (9)
ATCC 27,853	S (24)	S (21)	S (31)	S (29)	S (19)	S (21)	S (21)	S (23)	S (25)

^*a*^ ATM, aztreonam; IPM, imipenem; MEM, meropenem; CIP, ciprofloxacin; CTX, cefotaxime; CAZ, ceftazidime; AMK, amikacin; CEN, gentamicin; TZP, piperacillin/tazobactam.

^*b*^ R, resistant; S, susceptible; I, intermediate. Values in parentheses indicate inhibition zone diameters (mm) determined by the Kirby-Bauer method.

### Comparative genomic and phylogenetic analysis

Draft genomes of six *P. aeruginosa* clinical strains obtained by the Illumina platform revealed that they are homologous. Strain SCPa03-1 was selected as the representative and subjected to whole-genome sequencing via the Nanopore sequencer. Analysis of average nucleotide identity (ANI) analysis using the WGS data showed that SCPa03-1 had 99.41% identity to the genome of reference strain PAO1, but 98.76% identity to that of reference strain UCBPP-PA14 (abbreviated as PA14), indicating that these clinical strains are PAO1-like (16). Resfinder analysis indicated that they had identical antimicrobial resistance gene (ARG) profiles, mediating resistance to aminoglycosides (*aph(3')-IIb*), *β*-lactams (*bla*_PAO_, *bla*_OXA-486_), fosfomycin (*fosA*), chloramphenicol (*catB7*), and ciprofloxacin (*crpP*). MLST analysis revealed that six *P. aeruginosa* clinical strains belonged to ST274, which is an international epidemic high-risk clone that is frequently detected in clinical settings and commonly colonizes CF patients worldwide (17). To elucidate the phylogenetic relationships between the strains in our collection and other 293 ST274 isolates with available genomes from the Pseudomonas Genome Database (18), a phylogenetic tree based on 40,747 single nucleotide polymorphisms (SNPs) of their core genomes was constructed. Two groups (designated here as “Group A” and “Group B”) were clearly emerged from the tree (Fig. 1C). Six clinical *P. aeruginosa* strains from our collection clustered closely with the vast majority of isolates, falling into Group A. Strains in Group A were isolated from human, plant, and environmental samples collected across diverse geographical regions globally (Table S2), revealing clonal transmission of these strains in this group. Notably, our six strains share close genetic relatedness with two bile-derived isolates (strains 17122116 and 17122103) from China, suggesting that they share a common ancestor.

Virulence factor prediction showed that six *P. aeruginosa* strains in our collection had identical virulence gene profiles (Table S3). The detection of type III secretion system effector protein demonstrated the absence of *exoU* in these strains. Compared to the reference strain PAO1, six *P. aeruginosa* clinical strains carry fewer LPS O-antigen biosynthetic genes and have lost multiple genes involved in type IV pili biosynthesis (*fimT*, *pilA*, *pilV*, and *pilY2*), phenazine biosynthesis (*phzC2*, *phzD2*, *phzE2*, and *phzF2*), phospholipase D (*pldA*), type III secretion needle biogenesis (*pscE*), and pyoverdine biosynthesis (*pvdD*). Additionally, these *P. aeruginosa* clinical strains possess two genes enabling immune evasion that are absent in PAO1. The optimized virulence gene profiles in these clinical strains may facilitate bacterial infection establishment and adaptation to the host environment.

Genomic comparative analysis revealed high homology among six *P. aeruginosa* strains. The initial isolates SCPa03-1 and SCPa03-2 only differed by three mutations, and the four subsequently isolated (SCPa12-1, SCPa12-2, SCPa12-3, and SCPa12-4) differed by no more than 11 mutations compared with the previous two strains (Table 2). Compared to the ancestral isolate SCPa03-1, the four subsequent isolates all harbored nonsynonymous SNPs in the *cspA* (Cold shock protein), *algJ* (Alginate O-acetyltransferase), *epsL* (Sugar transferase), and *dppC* (ABC transporter permease) genes, in addition to the mutations in *gyrA* (DNA gyrase subunit A) and *prc* (Periplasmic tail-specific protease) that were also present in SCPa03-2. Specifically, both SCPa12-1 and SCPa12-2 harbored an additional nonsynonymous SNP in the RNA polymerase sigma factor-encoding gene *algU*. Additionally, SCPa12-1 had an amino acid substitution (V928I) in the cyclic di-GMP receptor MorA, and SCPa12-4 had G279C mutation in the 50S ribosomal protein L3 N (5) glutamine methyltransferase PrmB when compared to the ancestral isolate SCPa03-1.

**TABLE 2:** Single nucleotide polymorphism (SNP) differences compared with SCPa03-1

Strain	Gene	Annotation	Putative gene function
SCPa03-2	*gyrA*	A→G, N87D (AAC→GAC)	DNA gyrase subunit A
	*gyrB*	C→T, S466F (TCC→TTC)	DNA gyrase subunit B
	*prc*	(GACCAGCA)_2→1_, coding (1928-1935/1998 nt)	Tail-specific protease
SCPa12-1	*gyrA*	A→G, I174V (ATC→GTC), N116D (AAC→GAC), N87D (AAC→GAC)	DNA gyrase subunit A
	*prc*	(GACCAGCA)_2→1_, coding (1928-1935/1998 nt)	Tail-specific protease
	*cspA*	+A, coding (120/615 nt)	Cold shock protein
	*algU_2*	(CGACGCCCA)_1→2_, coding (134/582 nt)	RNA polymerase sigma-H factor
	*algJ*	Δ1 bp, coding (106/1176 nt)	Putative alginate O-acetylase AlgJ
	*epsL*	+AG, coding (339/600 nt)	Putative sugar transferase EpsL
	*dppC*	+57 bp, coding (302/855 nt)	Dipeptide transport system permease protein DppC
	*morA*	G→A, V928I (GTC→ATC)	Hypothetical protein
SCPa12-2	*gyrA*	A→G, I174V (ATC→GTC), N116D (AAC→GAC), N87D (AAC→GAC)	DNA gyrase subunit A
	*prc*	(GACCAGCA)_2→1_, coding (1928-1935/1998 nt)	Tail-specific protease
	*cspA*	+A, coding (120/615 nt)	Cold shock protein
	*algU_2*	(CGACGCCCA)_1→2_, coding (134/582 nt)	RNA polymerase sigma-H factor
	*algJ*	Δ1 bp, coding (106/1176 nt)	Putative alginate O-acetylase AlgJ
	*epsL*	+AG, coding (339/600 nt)	Putative sugar transferase EpsL
	*dppC*	+57 bp, coding (302/855 nt)	Dipeptide transport system permease protein DppC
SCPa12-3	*gyrA*	A→G, I174V (ATC→GTC), N116D (AAC→GAC), N87D (AAC→GAC)	DNA gyrase subunit A
	*prc*	(GACCAGCA)_2→1_, coding (1928-1935/1998 nt)	Tail-specific protease
	*cspA*	+A, coding (120/615 nt)	Cold shock protein
	*algJ*	Δ1 bp, coding (106/1176 nt)	Putative alginate O-acetylase AlgJ
	*epsL*	+AG, coding (339/600 nt)	Putative sugar transferase EpsL
	*dppC*	+57 bp, coding (302/855 nt)	Dipeptide transport system permease protein DppC
SCPa12-4	*gyrA*	A→G, I174V (ATC→GTC), N116D (AAC→GAC), N87D (AAC→GAC)	DNA gyrase subunit A
	*prc*	(GACCAGCA)_2→1_, coding (1928-1935/1998 nt)	Tail-specific protease
	*cspA*	+A, coding (120/615 nt)	Cold shock protein
	*algJ*	Δ1 bp, coding (106/1176 nt)	Putative alginate O-acetylase AlgJ
	*epsL*	+AG, coding (339/600 nt)	Putative sugar transferase EpsL
	*dppC*	+57 bp, coding (302/855 nt)	Dipeptide transport system permease protein DppC
	*prmB*	G→T, G279C (GGC→TGC)	50S ribosomal protein L3 glutamine methyltransferase

### Genetic basis underlying mucoid and non-mucoid phenotype

**Fig 2:**
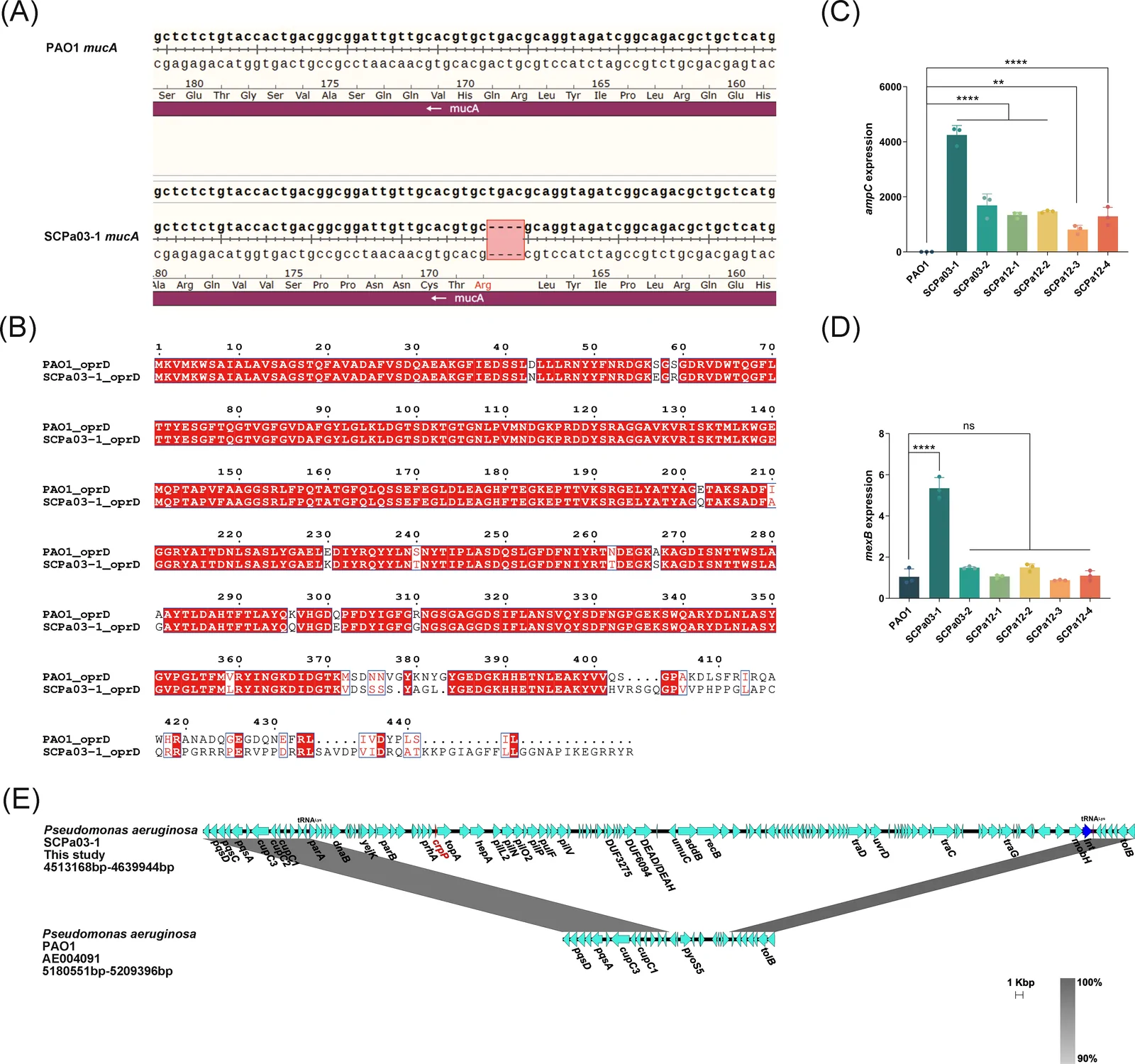
Genetic basis of mucoid phenotype and antimicrobial resistance in six *P. aeruginosa* isolates. (**A**) Sequence alignment of the *mucA* gene between SCPa03-1 and PAO1. A 4-bp nucleotide deletion (positions 503–506) in *mucA* of SCPa03-1 resulted in a frameshift mutation. The deleted 4-bp nucleotides are highlighted with a red border. (**B**) Amino acid sequence alignment of *oprD* between SCPa03-1 and PAO1. Sequences were aligned using Clustal X and visualized with ESPript 3.2. Identical residues are shown in white with a red background, while similar residues are shown in red. (**C**) qRT-PCR analysis of *ampC* gene expression. (**D**) qRT-PCR analysis of *mexB* gene expression. Data are shown as mean ± SD (*n* = 3) from three independent experiments performed in triplicate. Statistical analysis was performed using one-way ANOVA. **P < 0.01, ****P < 0.0001, and ns, not significant. (**E**) Genetic environment of the *crpP*-bearing genomic region in SCPa03-1 and comparison with the corresponding region in PAO1. Genes are denoted by arrows. The *crpP* gene, integrase gene, and tRNA^Lys^ gene are highlighted in red, blue, and pink. Remaining genes are indicated in cyan. Regions of >90% nucleotide identity are indicated by gray shading.

The six strains exhibited distinct colony phenotypes when grown on Congo red plates: SCPa03-1, SCPa12-1, and SCPa12-2 were non-mucoid, and the other three were mucoid (Fig. 1B). We therefore explored the genome-based molecular mechanisms underlying the mucoid or non-mucoid appearance. It has been established that the emergence of mucoid (alginate-overexpressing) morphotypes is commonly associated with mutations in the *mucA* gene (19). Nucleotide sequence analysis identified a frameshift mutation in the *mucA* gene, resulting from a 4-bp deletion at nucleotide positions 503–506, which yielded a truncated 167-amino acid MucA protein in these clinical strains (Fig. 2A). The mutated MucA is highly likely to have driven the mucoid phenotype in SCPa03-2, SCPa12-3, and SCPa12-4. In SCPa03-1, we detected an 8-bp insertion at nucleotide position 1914 of the *prc* gene, leaving a truncated 638-amino acid Prc protein. Prc is a 709-amino acid protease that is involved in degrading mutant forms of MucA, and mutations in the *prc* gene have been demonstrated to suppress alginate production in *mucA*-mutant strains of *P. aeruginosa* (19, 20), which plausibly explains why strain SCPa03-1 is non-mucoid. Regarding SCPa12-1 and SCPa12-2, the inserted 9 bp at nucleotide position 134 of the *algU* gene is most likely responsible for their non-mucoid appearance, given that this is the only genetic difference between the mucoid SCPa12-3 and non-mucoid SCPa12-2. The mutant AlgU protein likely failed to be released from the inhibitory binding of the MucA protein, thus suppressing the alginate overproduction induced by *mucA* mutation (21).

### Genetic basis underlying resistance to imipenem and ciprofloxacin

Given that all six *P. aeruginosa* strains exhibited resistance to imipenem, we investigated the molecular mechanisms underlying this resistance phenotype. Porin OprD of *P. aeruginosa* facilitates the diffusion of carbapenem antibiotics, particularly imipenem, and OprD-mediated carbapenem resistance can be achieved by the inactivation of this porin by frameshift mutation (22). Sequence analysis of OprD revealed 23 amino acid substitutions and two deletions in these six *P. aeruginosa* strains (Fig. 2B), while no mutations were detected in loop 2 or loop 3—domains known to be involved in the binding to and passage of imipenem (23). We further quantified the expression levels of chromosomal *β*-lactamase (*ampC*) and efflux pump gene *mexB*, which were also recognized as major contributors to carbapenem resistance in *P. aeruginosa* (24). Results showed that *ampC* was overexpressed in these six *P. aeruginosa* strains when compared to the reference strain PAO1 (Fig. 2C). The expression of *mexB* was significantly elevated in SCPa03-1, but not in the other five strains (Fig. 2D). It was reported that mutations in penicillin-binding protein FtsI reduce carbapenem susceptibility in *P. aeruginosa* (25); however, no substantive *fstI* mutation was detected in these six clinical strains. Collectively, *ampC* overexpression and *oprD* mutations could contribute partially to imipenem resistance in these six *P. aeruginosa* strains, while additional uncharacterized factors may also act synergistically to mediate this resistance phenotype.

Ciprofloxacin is one of the most widely used antibiotics to treat *P. aeruginosa* infections (26). *crpP* is considered a ciprofloxacin-modifying enzyme that was initially identified on a plasmid in *P. aeruginosa* (27), although subsequent studies have shown that it does not act as a fluoroquinolone-inactivating enzyme and is not consistently associated with ciprofloxacin resistance in *P. aeruginosa* clinical isolates (28, 29). In this study, we identified a *crpP*-like gene (95.96% identity) on the chromosome of these six *P. aeruginosa* strains. Genetic analysis showed that this *crpP*-like gene was also carried by an integrative and conjugative element (ICE) (Fig. 2E), as reported by a previous study (30). BLAST analysis revealed that the *crpP*-harboring ICE identified in our study was nearly identical to those from *P. aeruginosa* clinical strains WCHPA075019 (untypeable, Sichuan, China, 2017, CP028584) and HPHA13 (ST277, Shanghai, China, 2022, CP125780), suggesting that the *crpP*-like gene is likely to be widely disseminated among *P. aeruginosa* populations with different genetic backgrounds. In addition to *crpP*, ciprofloxacin resistance is frequently conferred by mutations in DNA gyrase (*gyrA* and/or *gyrB*) or topoisomerase IV (*parC* and/or *parE*) (26). We found that the six *P. aeruginosa* strains had at least one amino acid substitution in each of their *gyrA* and *gyrB* genes (Table 2). It is highly likely that the synergistic effects of chromosomal *gyrA*/*gyrB* mutations and the horizontal acquisition of *crpP*-harboring ICEs collectively contributed to ciprofloxacin resistance in SCPa03-1, SCPa03-2, SCPa12-2, and SCPa12-4. For strains SCPa12-1 and SCPa12-3, their failure to develop ciprofloxacin resistance (instead exhibiting intermediate susceptibility) may be attributed to differences in the expression levels of key efflux pumps. The exact mechanism requires further investigation.

### Characterization of virulence-associated phenotypes

**Fig 3:**
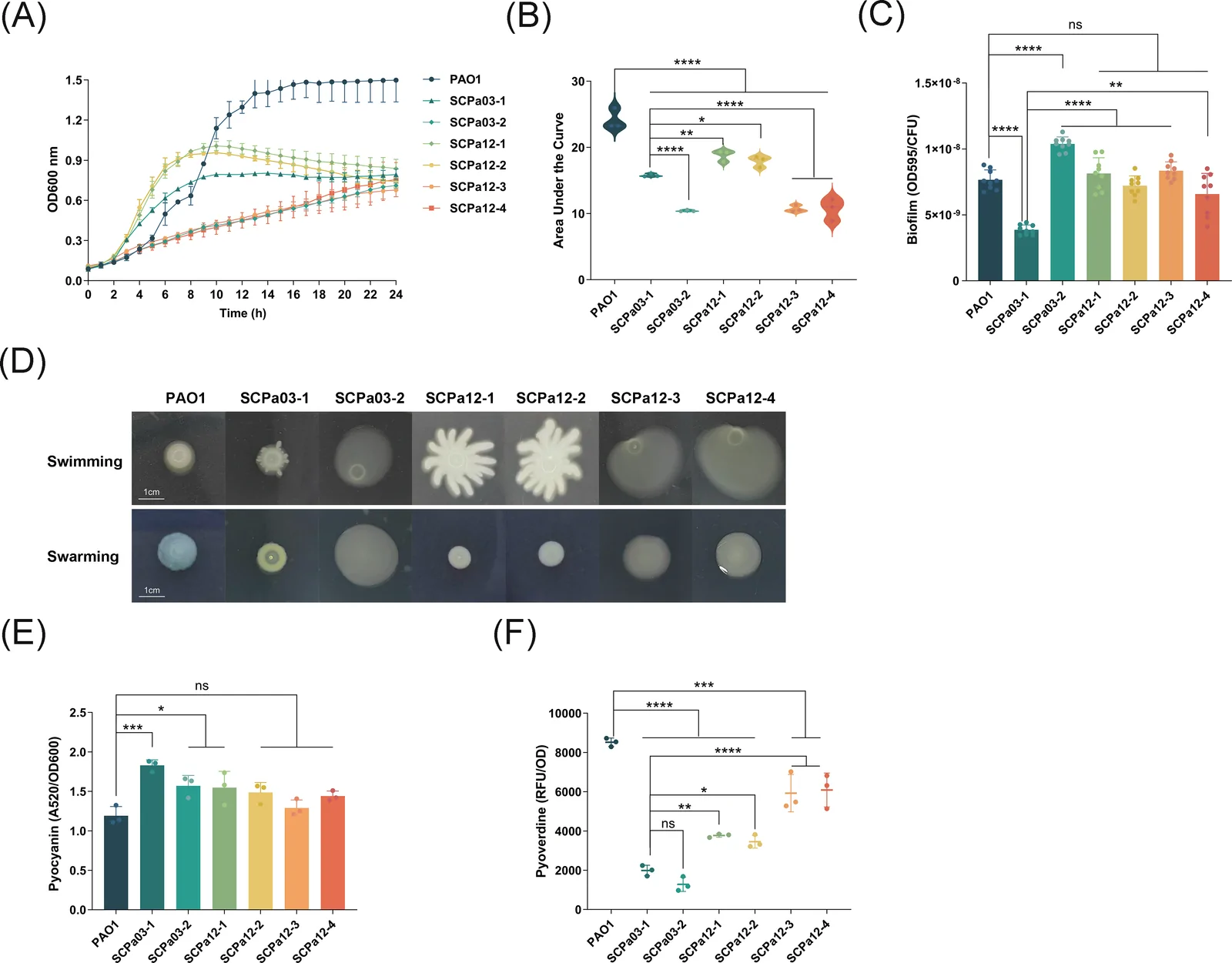
Virulence-associated phenotypes of six *P. aeruginosa* isolates. (**A**) Growth curves of *P. aeruginosa* strains. (**B**) Area under the growth curve (AUC) analysis of *P. aeruginosa* strains. AUC values were calculated from growth curves using GraphPad Prism 10.1.2 to evaluate bacterial growth capacity. (**C**) Quantification of biofilm formation. Biofilms were cultured in 96-well plates and stained with crystal violet at 24 h. (**D**) Representative images of swimming and swarming motility. The 1 cm scale is indicated. (**E**) Quantification of pyocyanin production. (**F**) Quantification of pyoverdine production. Data are shown as mean ± SD (*n* = 3) from at least three independent experiments performed in triplicate. Statistical analyses were performed using one-way ANOVA (**B, E, and F**) or Brown-Forsythe and Welch ANOVA tests followed by Dunnett’s T3 multiple comparisons test (**C**). *P < 0.05, **P < 0.01, ***P < 0.001, and ****P < 0.0001. ns, not significant.

To assess the growth kinetics of six clinical *P. aeruginosa* strains, their growth in LB broth was quantified via the area under the growth curve (AUC). Results showed that all six clinical isolates exhibited significantly decreased growth dynamics compared with PAO1 (all, *P* < 0.0001 vs PAO1; Fig. 3A and B). In comparison with non-mucoid SCPa03-1, the mucoid SCPa03-2, SCPa12-3, and SCPa12-4 exhibited significantly impaired growth capacity (all, *P* < 0.0001 vs SCPa03-1; Fig. 3A and B), suggesting a potential metabolic burden imposed by the mucoid phenotype.

Since *P. aeruginosa* commonly establishes chronic infections through biofilm formation (31), we further evaluated the 24-h biofilm formation of the six clinical strains. Compared with PAO1, SCPa03-1 formed significantly less biofilm, whereas SCPa03-2 formed significantly more (both *P* < 0.0001 vs PAO1). No significant differences were detected in the remaining four strains (Fig. 3C). Further comparative analysis revealed that all the other five strains displayed significantly enhanced biofilm formation than SCPa03-1 (all, *P* < 0.0001 vs SCPa03-1, except SCPa12-4, *P* = 0.0033. Figure 3C). Given that alginate is a key matrix component for biofilm formation, mutations in the *prc* gene of SCPa03-1 inhibited alginate production, which likely contributed to its substantial reduction in biofilm biomass (19, 20).

Flagellum-mediated swimming and swarming motility are key virulence-related behaviors in the pathogenesis of *P. aeruginosa* (32). Our data showed that SCPa03-1 displayed motility comparable to PAO1, whereas SCPa12-1 and SCPa12-2 had deficient swarming motility despite intact swimming ability (Fig. 3D). Impairment or modification of swimming motility represents one of the most frequent adaptive alterations associated with the formation of mucoid phenotypes in *P. aeruginosa* (33). However, in our study, the mucoid strains SCPa03-2, SCPa12-3, and SCPa12-4 exhibited robust swimming and also strong swarming motility (Fig. 3D), suggesting that they may possess a distinct dissemination advantage in the early phase of infection while also having the potential to establish persistent chronic infections.

Given that pyocyanin is a key virulence factor of *P. aeruginosa*, we measured its production in six clinical strains. Overall, pyocyanin production was significantly elevated in all six clinical isolates relative to the reference strain PAO1, although no statistical differences were observed in SCPa12-2, SCPa12-3, and SCPa12-4 (*P* = 0.0001, 0.0112, 0.0170, 0.0532, 0.8415, and 0.1165 vs PAO1, respectively; Fig. 3E). The increased pyocyanin production may help *P. aeruginosa* to withstand host-imposed stress during chronic infection.

Pyoverdine is the primary siderophore for iron acquisition and acts as a signal molecule to trigger virulence factor production (34). Compared with PAO1, the pyoverdine production was significantly reduced in all six clinical isolates, indicating impaired iron acquisition from the surrounding environment (SCPa03-1, SCPa03-2, SCPa12-1, and SCPa12-2 vs PAO1, *P* < 0.0001; SCPa12-3 vs PAO1, *P* = 0.0002; SCPa12-4 vs PAO1, *P* = 0.0004; Fig. 3F). Furthermore, compared with SCPa03-1 and SCPa03-2, four subsequent isolates (SCPa12-1, SCPa12-2, SCPa12-3, and SCPa12-4) exhibited elevated pyoverdine production, indicative of adaptive evolution in response to iron limitation (Fig. 3F). SNP analysis revealed a 57-bp insertion at nucleotide position 302 within the *dppC* gene in these four isolates, a genetic variant that was absent in SCPa03-1 and SCPa03-2 (Table 2). *dppC* encodes the permease DppC, a transmembrane component of the inner membrane ATP-binding cassette transporter complex DppBCDF that mediates dipeptide transport (35). The Dpp transport machinery has been demonstrated to be involved in heme iron utilization in *Escherichia coli* (36). The insertion mutation in *dppC* in this study may impede the bacterial utilization of exogenous iron sources such as heme, triggering the cellular sensing of iron limitation and the subsequent compensatory upregulation of pyoverdine synthesis (37). This provides a plausible explanation for the elevated pyoverdine production observed in these four isolates.

### Viability under host stress conditions

**Fig 4:**
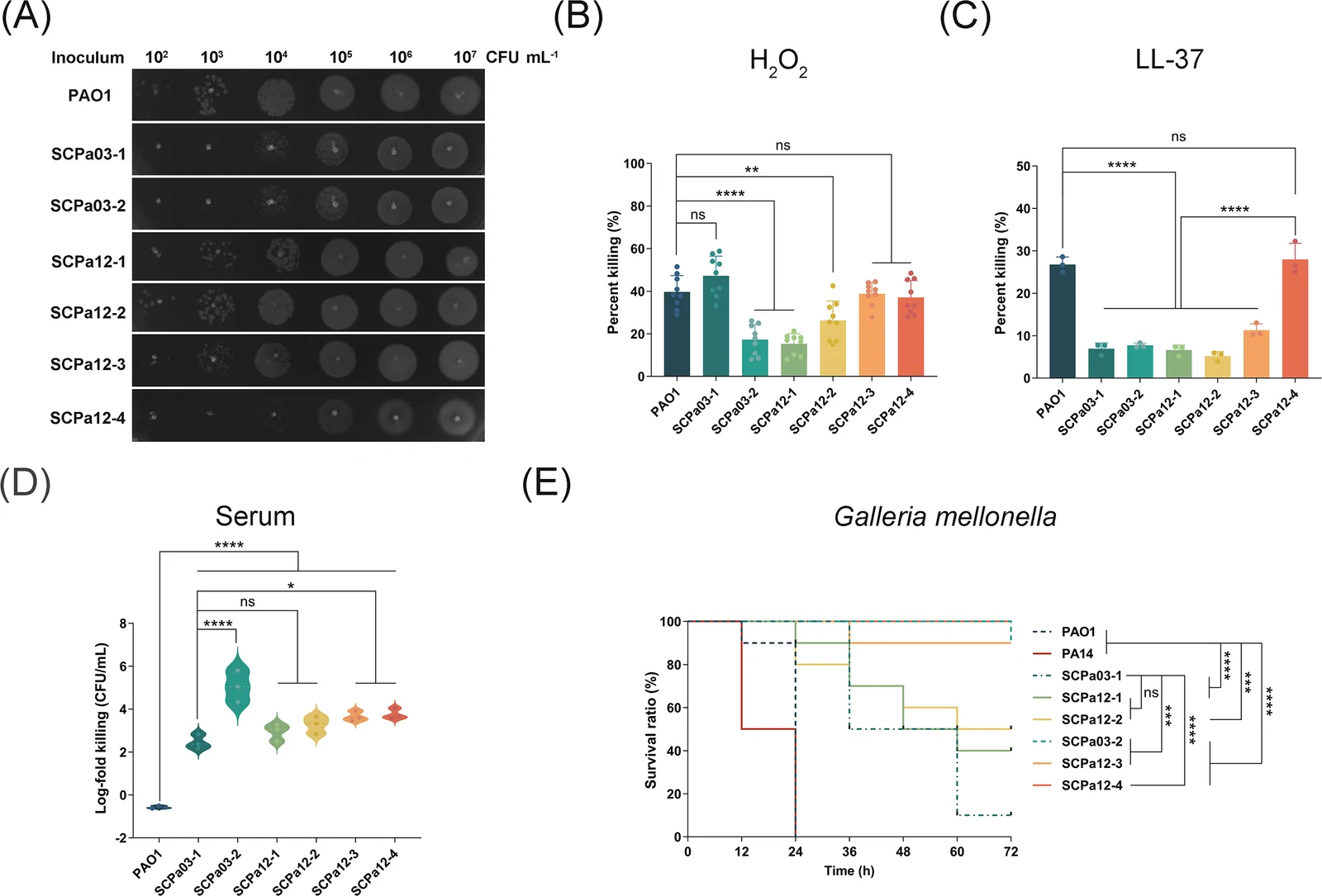
Host-stress tolerance and virulence of six *P. aeruginosa* isolates. (**A**) Growth of *P. aeruginosa* on iron-limited LB agar plates supplemented with 300 μM 2,2'-dipyridyl at different inoculum sizes. *P. aeruginosa* CFUs were quantified after H_2_O_2_ (**B**), LL-37 (**C**), or human serum (**D**) treatment, and killing rates or log-fold killing were determined. Bacteria treated with ddH_2_O or PBS served as controls. Data are shown as mean ± SD (*n* = 3) from three independent experiments performed in triplicate. Statistical analysis was performed using one-way ANOVA. (**E**) Survival ratio of *G. mellonella* larvae. A 10 μL volume of *P. aeruginosa* dilution (2 × 10^4^ CFU) or PBS (negative control) was injected into each larva. Survival was monitored every 12 h for 72 h post-infection. Statistical analysis was performed using the log-rank test (Kaplan–Meier method). *P < 0.05, **P < 0.01, ***P < 0.001, and ****P < 0.0001. ns, not significant.

We further evaluated the adaptability of six clinical *P. aeruginosa* strains in response to host-like environmental stress. Similar to the reference strain PAO1, SCPa12-1, SCPa12-2, and SCPa12-3 were able to form visible colonies even at a low inoculum (10²−10³ CFU/mL) on LB agar plates supplemented with 300 μM 2,2′-bipyridine, whereas SCPa03-1, SCPa03-2, and SCPa12-4 required an inoculum concentration of 10^4^ CFU/mL to form visible colonies on the iron-limited plate (Fig. 4A). In comparison with SCPa03-1 and SCPa03-2, the subsequent isolates SCPa12-1, SCPa12-2, and SCPa12-3 exhibited enhanced iron utilization capacity under iron-limiting conditions. This phenotype was consistent with the results of the pyoverdine production assay, with SCPa12-4 being an exception. Genomic analysis revealed that SCPa12-4 differs from SCPa12-3 by only a single SNP, namely, a G279C substitution in *prmB* (Table 2). *prmB* encodes a 50S ribosomal protein L3 glutamine methyltransferase that mediates post-translational methylation of L3 and contributes to ribosome assembly and function (38). This mutation in *prmB* may underlie the impaired growth of SCPa12-4 under iron-limited conditions.

After treatment with 50 mM H₂O₂, the killing rates of SCPa03-2 (17.28 ± 7.03%), SCPa12-1 (15.25 ± 4.79%), and SCPa12-2 (26.24 ± 9.22%) were significantly decreased compared with PAO1 (39.67 ± 7.69%) and SCPa03-1 (47.23 ± 9.23%; SCPa03-2 and SCPa12-1 vs PAO1, *P* < 0.0001; SCPa12-2 vs PAO1, *P* = 0.0020; Fig. 4B), indicating enhanced resistance to H₂O₂. To explore the underlying mechanism, we identified a 9-bp insertion at nucleotide position 134 of the *algU* gene specifically in SCPa12-1 and SCPa12-2 (Table 2). AlgU, an extracytoplasmic function sigma factor, acts as a key global regulator of environmental stress adaptation in *Pseudomonas* species and has been reported to positively regulate oxidative stress tolerance (39). The 9-bp insertion in *algU* may enhance the regulatory activity of AlgU on antioxidant defense pathways, thereby increasing oxidative stress resistance in SCPa12-1 and SCPa12-2.

After treatment with 50 μg/mL LL-37, an innate immune effector molecule, all the clinical isolates except SCPa12-4 displayed significantly decreased killing rates (<11.28 ± 1.52%) compared with PAO1 (26.74 ± 1.83%; all, *P* < 0.0001 vs PAO1; Fig. 4C), indicating enhanced resistance to LL-37. The identified G279C mutation in the *prmB* of SCPa12-4 may account for the attenuated resistance of SCPa12-4 to the antimicrobial peptide LL-37 (Table 2), as *prmB* was shown to be involved in mediating bacterial resistance to colistin, an antimicrobial peptide antibiotic (40).

After exposure to 80% human serum, the survival rate of PAO1 reached 376.3 ± 54.82%, whereas the survival rates of all six clinical isolates were below 1% (all, *P* < 0.0001 vs PAO1; Fig. 4D). These results indicate that serum resistance was markedly reduced in all six clinical isolates. Notably, the mucoid strains SCPa03-2, SCPa12-3, and SCPa12-4 exhibited significantly lower serum resistance than the non-mucoid strains SCPa03-1, SCPa12-1, and SCPa12-2 (SCPa03-2 vs SCPa03-1, *P* < 0.0001; SCPa03-2 vs SCPa12-3, *P* = 0.0184 and SCPa12-4 vs SCPa03-1, *P* = 0.0110; Fig. 4D), which is consistent with the previous study that the mucoid material of *P. aeruginosa* does not confer protection against serum bactericidal activity (41). The reduced serum resistance observed in the mucoid isolates suggests that evolutionary adaptation toward a colonization-associated mucoid phenotype may incur a fitness cost by compromising resistance to serum-mediated killing.

### Virulence in *Galleria mellonella*

A *G. mellonella* larvae infection model was employed to assess the virulence of these six clinical *P. aeruginosa* strains, with moderate-virulent strain PAO1 and high-virulent strains PA14 as references. An inoculum of 2 × 10^4^ CFU was administered to the larvae (*n* = 10 for each strain), and mortality was recorded within 72 h of injection. As a result, all larvae infected with PAO1 and PA14 died within 24 h post-infection, resulting in a survival rate of 0%. In contrast, the six clinical strain-infected groups exhibited significantly lower mortality after 24 h post-infection: the larval survival rates were 90% and 80% for SCPa12-1 and SCPa12-2, respectively, whereas the survival rates reached 100% for SCPa03-1, SCPa03-2, SCPa12-3, and SCPa12-4, indicating that these clinical strains displayed an attenuated virulence phenotype (all, *P* < 0.0001 vs PAO1 and PA14; except *P* = 0.0004 and 0.0002, SCPa12-2 vs PAO1 and PA14; Fig. 4E). After 72 h of infection, the survival rate of larvae infected with strains SCPa03-1, SCPa12-1, and SCPa12-2 dropped to 10%, 40%, and 50%, whereas those infected with strains SCPa03-2, SCPa12-3, and SCPa12-4 were 90%, 90%, and 100%, respectively. These results indicated that the mucoid isolates SCPa03-2, SCPa12-3, and SCPa12-4 exhibited significantly attenuated virulence compared with the non-mucoid isolates SCPa03-1, SCPa12-1, and SCPa12-2 (SCPa03-2 vs SCPa03-1, *P* = 0.0001; SCPa12-3 vs SCPa03-1, *P* = 0.0007 and SCPa12-4 vs SCPa03-1, *P* < 0.0001; Fig. 4E). These findings highlight that the mucoid phenotype is associated with reduced acute virulence and may promote adaptation to long-term colonization and the establishment of chronic infection.

## DISCUSSION

*P. aeruginosa* is one of the predominant pathogens causing chronic respiratory infections such as CF and COPD. Persistent selective pressures within the host, including immune responses and prolonged antimicrobial therapy, drive the adaptive evolutionary dynamics of this opportunistic pathogen (14). In this study, two *P. aeruginosa* strains were initially isolated from the sputum of a COPD patient. Five months later, an additional four strains were recovered from the same patient’s sputum, establishing a longitudinal framework to investigate the within-host microevolution of *P. aeruginosa* during the progression of chronic infection. All six isolates belonged to ST274, which is a globally epidemic high-risk clone closely associated with hospital-acquired infections and chronic airway colonization (3). *P. aeruginosa* ST274 is known as a globally disseminated multidrug-resistant clone that also exhibits a carbapenem-resistant phenotype (4). Consistent with this, all six isolates in our study showed imipenem resistance. While virulence factor profiles vary among ST274 isolates, this clone is linked to genotypes encoding core virulence determinants such as *exoS*, as well as adaptive traits relevant to biofilm formation that support persistent airway colonization (42). The convergence of multidrug resistance and adaptive virulence likely contributes to the ability of ST274 to establish long-term colonization in chronic respiratory infections.

In contrast to initial isolates SCPa03-1 and SCPa03-2, the four subsequent isolates (SCPa12-1, SCPa12-2, SCPa12-3, and SCPa12-4) exhibited an overall upregulation of pyoverdine biosynthesis, accompanied by an enhanced growth ability under iron-limiting conditions. Iron acquisition is essential for the survival of *P. aeruginosa*, as numerous core cellular functions and metabolic pathways are strictly iron dependent (13). However, the host restricts iron bioavailability through hepcidin-mediated iron sequestration, lipocalin-associated chelation, and other similar mechanisms, thereby limiting iron acquisition by invading pathogens (12). Thus, the enhanced pyoverdine biosynthesis in these four late-stage isolates confers a competitive advantage by enabling more efficient iron sequestration when competing with host iron-binding proteins (34). A 57-bp insertion in *dppC* was present in the four later isolates, but not in the initial ones. Given that *dppC* encodes a DppABC transporter permease that is involved in dipeptide and heme-bound iron uptake (36, 37), this mutation may impair exogenous iron acquisition, thereby increasing pyoverdine production in these four later isolates, which reflects adaptive remodeling of nutrient acquisition strategies during chronic infection. Notably, their enhanced iron acquisition further supports an evolutionary trajectory favoring improved resource sequestration and long-term persistence within the host, rather than a shift toward acute virulence phenotypes.

Of the six clinical isolates in this study, SCPa03-2, SCPa12-3, and SCPa12-4 were mucoid, whereas SCPa03-1, SCP12-1, and SCPa12-2 were non-mucoid despite harboring the identical *mucA* mutation. Specifically, in SCPa03-2, SCPa12-3, and SCPa12-4, the mucoid phenotype was primarily attributed to the *mucA* mutation, which led to the excessive production of alginate (19). By contrast, the *prc* mutation in SCPa03-1 and *algU* mutation in SCPa12-1 and SCPa12-2 appeared to suppress alginate overproduction in these *mucA*-mutant *P. aeruginosa* strains, thus resulting in the non-mucoid phenotype (19, 20).

In *P. aeruginosa*, *mucA* mutation suppresses the cAMP/Vfr signaling pathway by activating the AlgU regulon, thereby downregulating the expression of multiple invasive virulence factors independent of alginate overproduction (43). This mechanism may explain the attenuated virulence of all six *mucA*-mutant isolates in our study relative to the reference strain PAO1 in the *G. mellonella* infection model. The mucoid phenotype, characterized by elevated alginate biosynthesis and robust biofilm formation, is tightly coupled to the establishment of chronic host infections and is typically accompanied by global repression of quorum sensing systems, reduced expression of secreted virulence factors, and impaired growth kinetics (44, 45). Consistent with these established findings, three mucoid isolates (SCPa03-2, SCPa12-3, and SCPa12-4) displayed lower growth ability and attenuated virulence compared with the non-mucoid strains (SCPa03-1, SCP12-1, and SCPa12-2). While alginate is widely recognized as a component enabling mucoid *P. aeruginosa* to evade host innate immune effectors, studies have demonstrated that antibodies specific to this surface polysaccharide can enhance complement deposition and opsonophagocytosis, thereby promoting complement-dependent killing (46). This mechanism may partially explain the reduced serum resistance of the mucoid isolates SCPa03-2, SCPa12-3, and SCPa12-4 observed in this study. Our results collectively suggest that the mucoid phenotype, together with its associated phenotypic alterations in growth, virulence, and serum resistance, represents an adaptive response to environmental and host-derived pressures, reflecting a fundamental trade-off in bacterial survival strategies that favors long-term colonization and chronic adaptation. In contrast, these non-mucoid strains are more closely associated with acute, hypervirulent infection patterns and exhibit enhanced growth and invasive potential.

Mucoid strains (SCPa03-2, SCPa12-3, and SCPa12-4) in this study were resistant to most tested *β*-lactam antibiotics but sensitive to meropenem, while non-mucoid isolates (SCPa03-1, SCP12-1, and SCPa12-2) were resistant to all tested *β*-lactam antibiotics, including meropenem. This finding that three mucoid isolates remained relatively susceptible to meropenem is consistent with previous studies, where mucoid variants exhibited higher antibiotic susceptibility than their non-mucoid counterparts (47, 48). Rather than imposing a selective disadvantage, the retained antibiotic susceptibility in alginate-overproducing isolates is likely sustained *in vivo* via a group protection mechanism mediated by coexisting antibiotic-resistant, non-mucoid subpopulations residing within the biofilm (47). This group protection concept aligns with the “insurance hypothesis,” which proposes that *P. aeruginosa* spontaneously generates genetic and functional diversity within biofilms, and these heterogeneous variants act synergistically to enhance the environmental stress resistance of the biofilm community (49). Supporting this view, mixed mucoid and non-mucoid *P. aeruginosa* populations have been shown to gain collective fitness via shared protective factors (e.g., alginate and catalase) under host-derived stresses (50). Collectively, our findings confirm that the coexistence of mucoid and non-mucoid strains within the host may constitute a synergistic adaptive strategy that enhances population-level tolerance to immune and antibiotic pressures, thereby facilitating persistence during chronic infection.

The six *P. aeruginosa* clinical isolates recovered from a single COPD patient in this study exhibited clonal relatedness yet displayed substantial heterogeneity in mucoid phenotype, antimicrobial susceptibility, and virulence, suggesting that they had undergone pronounced within-host adaptive diversification during persistent colonization. Previous longitudinal genomic studies of CF patients have demonstrated that persistent *P. aeruginosa* clones progressively accumulate adaptive mutations that modify regulatory, metabolic, resistance, and extracellular virulence pathways, resulting in extensive genotypic and phenotypic heterogeneity (51). Although COPD and CF differ in airway pathophysiological environments, as well as in the colonization persistence and population epidemiological profiles of *P. aeruginosa* (15, 52), the substantial phenotypic divergence among clonally related isolates observed within merely 5 months in this study indicates that strains persistently colonizing COPD airways may share partially adaptive evolutionary paths with chronically colonizing isolates from CF patients. This aligns with previous studies of *P. aeruginosa* within-host microevolution in COPD. Martínez-Solano et al. demonstrated that individual *P. aeruginosa* clones persist within the airways of patients with COPD over several years, diversifying into phenotypically heterogeneous, coexisting subpopulations (52). Subsequent whole-genome sequencing analyses by Eklöf et al., based on a prospective cohort of 23 patients, further validated that *P. aeruginosa* predominantly establishes persistent monoclonal colonization in COPD airways, accompanied by the enrichment of high-frequency mutations in genes associated with antimicrobial resistance and chronic infections (53). By integrating genomic and phenotypic analyses of clonally related isolates from a single patient, our study provides complementary isolate-level insights into short-term within-host diversification beyond prior phenotypic or genomic studies.

In conclusion, we systematically characterized six ST274 *P. aeruginosa* strains isolated from a COPD patient, identifying within-host adaptive diversification via genomic and phenotypic analyses. Divergent mucoidy, antimicrobial resistance, stress tolerance, and virulence traits reflect an evolutionary trade-off that may drive persistent infection and complicate treatment. These findings provide further insight into the short-term adaptation of imipenem-resistant *P. aeruginosa* in COPD and may contribute to a better understanding of the management challenges posed by drug-resistant respiratory infections.

## MATERIALS AND METHODS

### Bacterial strains, isolation, and culture conditions

Six *P. aeruginosa* isolates were sequentially collected from sputum samples of the same COPD patient. The sputum samples were separately inoculated onto blood agar plates, chocolate agar plates, and MacConkey agar plates (all provided by Antu Biotechnology, China) for cultivation. Blood agar plates and chocolate agar plates were incubated at 35°C in a 5% CO₂ incubator, while MacConkey agar plates were incubated at the same temperature in a conventional incubator. Bacterial growth was observed after 24 and 48 h of incubation, respectively. Suspected colonies were purified by subculturing on blood agar plates until morphologically uniform colonies with no obvious variation were obtained, and a single colony was then randomly selected from the purified culture. Species identification of all isolates was performed via matrix-assisted laser desorption/ionization time-of-flight mass spectrometry (MALDI-TOF/MS). Unless otherwise specified, all *P. aeruginosa* strains were inoculated into LB liquid medium and cultured overnight at 37°C with shaking at 220 rpm.

### Antimicrobial susceptibility testing

The antimicrobial susceptibility of these strains was determined using the Kirby-Bauer disk diffusion method for the following antimicrobial agents: aztreonam (30 μg), imipenem (10 μg), meropenem (10 μg), ciprofloxacin (5 μg), ceftazidime (30 μg), cefotaxime (30 μg), amikacin (30 μg), gentamicin (30 μg), and piperacillin/tazobactam (36 μg) according to the Clinical and Laboratory Standards Institute (CLSI) guideline (54). The log-phase bacterial culture was adjusted to a 0.5 McFarland (~1.5 × 10^8^ CFU/mL), and 100 μL of the bacterial suspension was plated on the Mueller-Hinton agar plates (Solarbio, Beijing, China). After incubation at 37°C for 16–18 h with antibiotic disks, the inhibition zone diameters were measured. *P. aeruginosa* ATCC 27853 served as a quality control strain for susceptibility testing. The interpretation of the results was based on the CLSI guideline.

### Whole-genome sequencing and bioinformatic analysis

Genomic DNA was extracted using the QIAamp DNA Mini Kit (Qiagen). Purified DNA was subjected to WGS on an HiSeq 2000 sequencer (Illumina, San Diego, CA), with a 150 bp paired-end library by the Tsingke Biotech (Beijing, China). Raw sequencing reads were processed for quality trimming via Trimmomatic v0.38 (55), and clean reads were subsequently assembled into draft genomic sequences using SPAdes v3.12.0 (56). SCPa03-1 was further subjected to WGS using the long-read MinION Sequencer (Nanopore, Oxford, UK) by the Novogene Biotech (Beijing, China). Genome assembly of the second- and third-generation sequencing data was accomplished using Unicycler v0.5.0 (57). Genomic annotation was carried out using Prokka software v1.14 (58). Analysis of average nucleotide identity was performed by the ANI Calculator (16). Antibiotic resistance genes (ARGs) and virulence genes were predicted via ResFinder database (59) and Virulence Factor Database (VFDB) (60), respectively. Single nucleotide polymorphism analysis was performed by aligning the genome sequence of SCPa03-1 with those of the other five isolates using Breseq version 0.39.0 (61). Furthermore, the SnapGene tool was applied for gene sequence alignment (GSL Biotech, http://www.snapgene.com/).

### Phylogenetic tree construction

Phylogenetic analysis was performed based on core-genome SNPs as previously described (62). Briefly, genome sequences of the six strains in this study and 293 ST274 isolates retrieved from the Pseudomonas Genome Database (https://www.pseudomonas.com/) were annotated with Prokka v1.14. The resultant GFF3 files were imported into Roary to generate core-genome sequence alignments. SNPs were identified and extracted using snp-sites v2.3.2, and a maximum-likelihood phylogenetic tree was constructed from these SNP data via FastTree v2.1.10 under the GTR model.

### Quantitative reverse transcription PCR (qRT-PCR)

The expression levels of *mexB* and *ampC* were quantified by qRT-PCR, following the method described previously (63). Strains were cultured to the logarithmic phase, with an optical density at 600 nm (OD₆₀₀) of 0.4–0.6. Total RNA extraction was performed in accordance with the protocols of Bacterial RNA Kit (GBCBIO Technologies, China). Subsequently, the RNA was immediately reverse-transcribed into cDNA via two steps: genomic DNA removal and reverse transcription, following the instructions of the PrimeScript RT Reagent Kit with gDNA Eraser (TaKaRa, Japan). The cDNAs obtained were stored at −20°C until use. The PCR reaction was performed on a Real-Time PCR Detection System (BIOER, Hangzhou, China; Model FQD-96A). Following serial dilution of the cDNA, a standard curve was generated to validate each primer’s amplification efficiency. Relative quantification was obtained using the 2^−ΔΔCt^ method, with the *rpsL* gene as the housekeeping gene. The mRNA transcription level of the target gene was expressed as a value relative to that of *P. aeruginosa* PAO1. An effect on gene expression was considered significant when the corresponding ratios were > 3 or <2 for *mexB* and > 5 or <10 for *ampC* (64). The experiment was repeated at least three times in triplicate, and the primers used for qRT-PCR are shown in Table S1.

### Growth assay

*P. aeruginosa* from overnight cultures was sub-cultured at a 1:100 dilution in LB medium and cultivated to the logarithmic phase. The log-phase bacterial culture was then adjusted to an OD_600_ of 0.1 (~1 × 10⁸ CFU/mL), and 200 μL of the suspension was added to each well of a flat-bottomed 96-well microplate (Corning, USA). Plates were incubated at 37°C for 24 h, and OD_600_ was measured every 60 min using a microplate reader (BioTek Synergy H1, USA), with shaking prior to each reading. The experiment was repeated at least three times in triplicate.

### Biofilm assay

A biofilm formation assay was determined using crystal violet staining on a 96-well microtiter plate with some modifications (65). The log-phase bacterial culture was adjusted to an OD_600_ of 0.1 (~1 × 10⁸ CFU/mL). After a 100-fold dilution, 200 μL of this bacterial suspension was added to a flat-bottomed 96-well microplate and incubated at 37°C for 24 h, with LB medium as a negative control. Subsequently, 100 μL of bacterial suspension was collected from one well per strain, serially diluted, and plated on agar for colony counting. The remaining culture in the other wells was removed, and the wells were washed three times with pure water. After drying, the adherent biofilms were fixed with methanol for 15 min and then dried again. The wells were subsequently stained with 0.1% crystal violet for 15 min, washed three times with pure water, and dried. Finally, the bound dye was eluted with 200 μL of 36% glacial acetic acid for 15 min, and the absorbance of the eluate was measured at 595 nm using a microplate reader. Biofilm formation was quantified as the ratio of OD₅₉₅ to CFU. The experiment was repeated at least three times in triplicate.

### Motility assay

Swimming and swarming assays were employed to assess the motility of *P. aeruginosa*, according to the method with some modifications (66). Swimming agar medium (tryptone: 10 g/L^−1^, NaCl: 5 g/L^−1^, agar powder: 0.3 g/L^−1^) and swarming agar medium (LB Broth Powder: 8 g/L^−1^, glucose: 2.5 g/L^−1^, agar powder: 0.5 g/L^−1^) were prepared. Plates were point-inoculated with 2 μL of overnight cultures grown on LB agar. Plates were incubated at 37°C, and growth was assessed at 18 h for swimming and 24 h for swarming.

### Pyocyanin assay

Pyocyanin production was determined by extraction from culture supernatants and quantified using a microplate reader (67). Strains were inoculated from the plates into LB medium, cultured overnight at 37°C, and their OD₆₀₀ values were then measured. Subsequently, the bacterial culture was centrifuged at 12,000 × *g* for 2 min, and 3 mL of the resulting supernatant was collected. Then, 1.8 mL of chloroform was added and mixed thoroughly. Next, 0.5 mL of 0.2 mol/L HCl was added to the 1.5 mL chloroform layer and vortexed vigorously. After centrifugation, 200 µL of the upper layer was collected to measure its absorbance at 520 nm. Pyocyanin concentration (μg/mL/OD₆₀₀) was calculated as (A₅₂₀ × 17.072)/OD₆₀₀. The experiment was repeated at least three times in triplicate.

### Pyoverdine assay

Pyoverdine production of *P. aeruginosa* was assayed by growing bacteria in King’s B medium, as described previously (68). Strains were inoculated from the plates into 3 mL King’s B medium and cultured at 37°C with shaking at 220 rpm for 48 h. After incubation, the OD₆₀₀ of the bacterial culture was measured. The culture was then centrifuged at 5,000 × *g* for 10 min, and the supernatant was collected. Fluorescence emission intensity of the supernatant was measured using a microplate reader with excitation/emission wavelengths of 400/460 nm. Pyoverdine production was quantified as the ratio of sample fluorescence intensity (RFU) to bacterial culture OD₆₀₀ (RFU/OD) for standardized data analysis. The experiment was repeated at least three times in triplicate.

### Iron-limiting spot assay

The iron acquisition capacity of *P. aeruginosa* was evaluated using an iron-restricted spotting assay (69). Bacteria were grown in LB broth to stationary phase, adjusted to approximately 10⁷ CFU/mL, serially diluted in phosphate-buffered saline (PBS), and 5 μL aliquots were spotted onto LB agar supplemented with 300 μM 2,2′-dipyridyl. The plates were then incubated at 37°C for 12 h, and bacterial growth was observed.

### Bacterial killing assay

The host-stress assay was performed with some modifications as previously described (50). Overnight bacterial cultures were subcultured 1:100 in LB medium and grown to the mid-logarithmic phase (approximately an OD₆₀₀ of 0.5 or 2 × 10⁸ CFU/mL). For the H_2_O_2_ killing assay, 300 μL of log-phase bacterial culture was centrifuged at 10,000 × *g* for 5 min. The cell pellet was resuspended in an equal volume of either 50 mM H₂O₂ or ddH₂O (negative control) and incubated at 37°C with rotation for 15 min. After that, cells were washed once with ddH₂O, serially diluted, and plated on agar for colony counting. For the LL-37 killing assay, log-phase bacterial culture was pelleted and resuspended in PBS. The bacterial suspension was then mixed 1:1 with either 50 μg/mL LL-37 or PBS (negative control) and incubated at 37°C for 1 h, followed by serial dilution and plating on agar for colony counting. The bacterial killing rate = [1−(CFU treated with H_2_O_2_ or LL-37)/(CFU treated with ddH₂O or PBS)] × 100%.

For the serum complement-mediated killing assay, log-phase bacterial cultures were centrifuged at 6,000 × *g* for 5 min to collect pellets, which were then resuspended in PBS and adjusted to an OD₆₀₀ of 0.1 (~1 × 10⁸ CFU/mL). Subsequently, the bacterial suspension was diluted 10-fold with PBS, followed by treatment with human serum from at least five healthy volunteers at a 1:4 (vol/vol) ratio, resulting in an 80% serum concentration in a final volume of 120 μL. PBS was used as the negative control. The mixture was incubated at 37°C for 120 min, after which samples were serially diluted and plated on agar for colony counting. CFU values were expressed as log₁₀ fold changes. Log-fold killing (CFU/mL) = log_10_(CFU with PBS) − log_10_(CFU with serum). Each experiment was performed at least three times with triplicate samples.

### *Galleria mellonella* infection assays

The virulence potential of *P. aeruginosa* strain was assessed using *G. mellonella* larvae (weighing 250 to 350 mg, Beijing Anxinkang Technology Co., Ltd., Beijing, China) as described previously (67). Log-phase bacterial cultures were plated on LB agar plates and incubated overnight at 37°C. Bacterial cells were then scraped from the plates and resuspended in PBS to an OD₆₀₀ of 1.0 (~2 × 10⁹ CFU/mL), followed by a 1:1,000 dilution in PBS. After that, 10 μL of *P. aeruginosa* dilution (2 × 10^4^ CFU) was injected into the hemocoel of each larva using a 25 μL Hamilton syringe. Ten larvae were infected per bacterial strain. Three larvae injected with 10 μL of sterile PBS served as the control group. The larvae were incubated at 37°C, and mortality was monitored every 12 h for 72 h. Dead larvae are inert and usually turn black.

### Statistical analysis

All data were analyzed using GraphPad Prism 10.1.2 software and expressed as means ± standard deviation (SD). For the biofilm assay, data were normally distributed but exhibited unequal variances. Therefore, statistical significance was assessed using the Brown-Forsythe and Welch ANOVA tests, followed by Dunnett’s T3 multiple comparisons test. For the *Galleria mellonella* killing assays, survival curves were generated using the Kaplan–Meier method, and significance was determined by the log-rank test. For all other experiments, their data met the assumptions of normality and homogeneity of variance, and significance was analyzed using one-way ANOVA. Significance levels were denoted as follows: *P < 0.05, **P < 0.01, ***P < 0.001, and ****P < 0.0001; ns, not significant.

## Data Availability

The whole-genome sequences of SCPa03-1, SCPa03-2, SCPa12-1, SCPa12-2, SCPa12-3, and SCPa12-4 have been deposited into the public GenBank database under project no. PRJNA1435050.

## SUPPLEMENTAL MATERIAL

Supplemental legends spectrum.01358-26-s0001.docx Legends for Tables S1 to S3.

Table S1 spectrum.01358-26-s0002.docx Primer sequences for qRT-PCR.

Table S2 spectrum.01358-26-s0003.xlsx Basic information of Group A and Group B *Pseudomonas aeruginosa* ST274 isolates from the phylogenetic analysis.

Table S3 spectrum.01358-26-s0004.xls Virulence gene profiles of six clinical *Pseudomonas aeruginosa* isolates.

ASM does not own the copyrights to Supplemental Material that may be linked to, or accessed through, an article. The authors have granted ASM a non-exclusive, world-wide license to publish the Supplemental Material files. Please contact the corresponding author directly for reuse.
