# Supplementary material for: Genomic and phenotypic adaptation of imipenem-resistant Pseudomonas aeruginosa during chronic infection in a COPD patient
Source: Microbiol Spectr. 2026 Aug 20;14(10):e01358-26. doi: 10.1128/spectrum.01358-26 (PMC13689455; doi:10.1128/spectrum.01358-26)
Supplement: Supplementary file 1 [file spectrum.01358-26-s0001.docx]

**Table S1. Primer sequences used for quantitative reverse transcription PCR (qRT-PCR).** Forward (F) and reverse (R) primer sequences of *mexB*, *ampC*, and *rpsL* are presented in the 5′–3′ orientation. Expected product lengths are indicated in base pairs (bp). Primers for *mexB* and *rpsL* were adopted from reference 1 and reference 2, respectively, whereas the *ampC* primers were designed in the present study.

**Table S2. Basic information on *Pseudomonas aeruginosa* ST274 isolates used for phylogenetic analysis.** The table contains strain designation, geographic origin, host, associated clinical conditions, and isolation source. Isolates are divided into phylogenetic Group A and Group B in separate worksheets. “Unknown” indicates that the relevant information is unavailable.

**Table S3. Virulence gene profiles of six clinical *Pseudomonas aeruginosa* isolates.** The table summarizes the classification of virulence factor (VF), virulence factors, corresponding related genes, and homologous gene loci predicted in each isolate, alongside reference loci from *P. aeruginosa* PAO1 and *P. aeruginosa* UCBPP-PA14. Data sheets for individual isolates are presented on separate worksheets. Tables were retrieved from the VFDB database ([http://www.mgc.ac.cn/VFs/](https://link.wtturl.cn/?target=http%3A%2F%2Fwww.mgc.ac.cn%2FVFs%2F&scene=im&aid=582478&lang=zh)).
