# Supplementary material for: Genomic and phenotypic adaptation of imipenem-resistant Pseudomonas aeruginosa during chronic infection in a COPD patient
Source: Microbiol Spectr. 2026 Aug 20;14(10):e01358-26. doi: 10.1128/spectrum.01358-26 (PMC13689455; doi:10.1128/spectrum.01358-26)
Supplement: Supplementary file 2 [file spectrum.01358-26-s0002.docx]

**Table S1 Primer sequences for qRT-PCR.**

| **Primer** | **Sequence (5'- 3')** | **Procduct length (bp)^a^** | **Reference** |
| --- | --- | --- | --- |
| *mexB*-F | CAAGGGCGTCGGTGACTTCCAG | 273 | （1） |
| *mexB*-R | ACCTGGGAACCGTCGGGATTGA |  |  |
| *ampC*-F | GCTGGGAAGCCTACGACTGG | 240 | This study |
| *ampC*-R | CCCGCTCGGCATTGGGATAG |  |  |
| *rpsL*-F | GCAAGCGCATGGTCGACAAGA | 201 | （2） |
| *rpsL*-R | CGCTGTGCTCTTGCAGGTTGTGA |  |  |

^a^bp, base pair.

1. Yin L, Bao Z, He L, Lu L, Lu G, Zhai X, et al. Virulence factors, molecular characteristics, and resistance mechanisms of carbapenem-resistant *Pseudomonas aeruginosa* isolated from pediatric patients in Shanghai, China. *BMC Microbiol* (2025) 25(1):130. doi: 10.1186/s12866-025-03856-1.

2. Dumas JL, van Delden C, Perron K, Köhler T. Analysis of antibiotic resistance gene expression in Pseudomonas aeruginosa by quantitative real-time-PCR. *FEMS Microbiol Lett* (2006) 254(2):217-25. doi: 10.1111/j.1574-6968.
